# Antibacterial Activity on Orthopedic Clinical Isolates and Cytotoxicity of the Antimicrobial Peptide Dadapin-1

**DOI:** 10.3390/ijms24010779

**Published:** 2023-01-02

**Authors:** Davide Campoccia, Lucio Montanaro, Stefano Ravaioli, Valentina Mariani, Giulia Bottau, Andrea De Donno, Carla Renata Arciola

**Affiliations:** 1 Laboratorio di Patologia delle Infezioni Associate all’Impianto, IRCCS Istituto Ortopedico Rizzoli, Via di Barbiano 1/10, 40136 Bologna, Italy; 2 Department of Medical and Surgical Sciences (DIMEC), University of Bologna, Via San Giacomo 14, 40126 Bologna, Italy

**Keywords:** Dadapin-1, antimicrobial peptides, implant infections, anti-infective biomaterials, orthopaedic infections, cytotoxicity

## Abstract

In orthopedic surgery, biomaterial-associated infections represent a complication of serious concern. Most promising strategies to prevent these infections currently rely on the use of anti-infective biomaterials. Desirably, in anti-infective biomaterials, the antibacterial properties should be achieved by doping, grafting, or coating the material surfaces with molecules that are alternative to conventional antibiotics and exhibit a potent and highly specific activity against bacteria, without altering the biocompatibility. Antimicrobial peptides (AMPs) are among the most interesting candidate molecules for this biomaterial functionalization. Here, the potential expressed by the recently discovered peptide Dadapin-1 was explored by assaying its MIC, MBIC and MBC on clinical strains of relevant bacterial species isolated from orthopedic infections and by assessing its cytotoxicity on the human osteoblast-like MG63 cells. When appropriately tested in diluted Mueller Hinton Broth II (MHB II), Dadapin-1 exhibited significant antibacterial properties. MIC values were in the range of 3.1–6.2 µM for the gram-positive bacteria *Staphylococcus aureus*, *Staphylococcus epidermidis*, and *Staphylococcus warneri*, and 12.4–24.9 µM for the gram-negative bacteria *Escherichia coli* and *Pseudomonas aeruginosa*. Interestingly, the peptide was found non-cytotoxic, with an IC50 exceeding the highest concentration tested of 179 µM. Overall, Dadapin-1 expresses considerable potential for future application in the production of anti-infective biomaterials.

## 1. Introduction

Infection is largely recognized as one of the most serious complications associated with the use of biomaterials in medicine. In orthopedics, infections often compromise the success of prosthetic surgery, leading to loss of osseointegration, loosening and failure of the implant [1,2]. Prosthetic infections are particularly difficult to diagnose and treat and often require two-step implant replacement procedures [3]. Conventional antibiotic treatments exhibit limited efficacy in curing biomaterial-associated infections. This is not only due to the spread of multiresistant bacterial strains, but also to the ability of common etiologic agents that cause these infections to form biofilms. While planktonic bacteria tend to be vulnerable to antibiotic treatments and usually are successfully treated, sessile bacteria that have adhered to prosthetic surfaces form protective biofilms, which are highly tolerant to most medical treatments and skew host immune defenses [4,5]. Thus, hindering bacterial adhesion on biomaterial surfaces has emerged as the primary strategy to prevent implant infections. We have previously described the wealth of biomaterials technologies that can be utilized to obtain infection-resistant surfaces [6]. In this regard, effective anti-infective biomaterials can be achieved by functionalizing the material surfaces with antibacterial molecules. Ideally, the employed antibacterial molecules should be alternative to conventional antibiotics, exhibit a broad spectrum of activity extended to multidrug-resistant strains, and have a potent and selective action on bacteria, without causing toxic effect or compromising the biocompatibility of the biomaterial.

Over the years, a multitude of substances (including metals, nanomaterials, disinfectants, phytocompounds and so on) have been found to possess bacteriostatic or bactericidal properties. Nonetheless, only a very few of them have been demonstrated to be safe and free from cytotoxic activity at the bactericidal concentration.

Antimicrobial peptides (AMPs) are emerging as being among the most versatile and interesting candidate molecules for therapeutic [7,8] and biomedical applications such as biomaterials functionalization [9,10,11,12]. Nowadays, over 3400 AMPs have been reported and characterized and a few of them have already been approved by the U.S. Food and Drug Administration (FDA) as peptide therapeutics [13]. Starting from the sequence of existing natural AMPs, new peptide sequences can be designed and modified, achieving an almost unlimited panel of candidate therapeutic molecules. Moreover, antimicrobial peptides appear very promising for the design of novel classes of therapeutic agents by reason of their scarce ability to induce resistance in pathogenic microorganisms [14].

This study was conducted on the antimicrobial peptide Dadapin-1, a new polycationic peptide (charge +5) recently developed and described by Rončević et al. (2019) [15]. Peptide sequences accessible in the Database of Anuran Defense Peptides (DADP) were extracted and subjected to the Mutator tool in order to design new antimicrobials with high predicted specificity index (SI = HC_50_/MIC; HC_50_, concentration causing 50% hemolysis; MIC, minimal inhibitory concentration). During this process, Dadapin-1 emerged from a panel of eight selected AMPs as the most promising one, showing poor haemolytic properties, while being capable of disrupting the bacterial membrane even at sub-MIC concentrations [15]. Dadapin-1 was found to show a clear transition to a partly helical structure in the presence of trifluoroethanol. Nonetheless, the helix content was estimated to be <50%. A strong contribution by the disordered structure (>70%) was reported in the presence of anionic SDS micelles and 1,2-dipalmitoyl-sn-phosphatidylglycerol, suggesting that it interacts with bacterial membranes only as a partially helical peptide [15].

This study aimed at exploring more in depth the potential of this novel AMP as a bactericidal substance, alternative to conventional antibiotics, for the future development of anti-infective functionalized biomaterials in orthopaedics. For this reason, Dadapin-1 antibacterial properties were tested on ten different bacterial strains, including nine clinical isolates from orthopedic infections. The panel of strains included five gram-positive and two gram-negative species, representative of etiological agents frequently causing post-surgical implant-related orthopaedic infections [16]. Moreover, Dadapin-1′s still unexplored cytotoxicity on in vitro cultured human cells was assayed on a reference strain of human osteoblast-like cells, the MG63 cells, often utilized to assess the biocompatibility in vitro of biomaterials for orthopedic prostheses.

## 2. Results

### 2.1. Dadapin-1 Antimicrobial Properties

**Figure 1:**
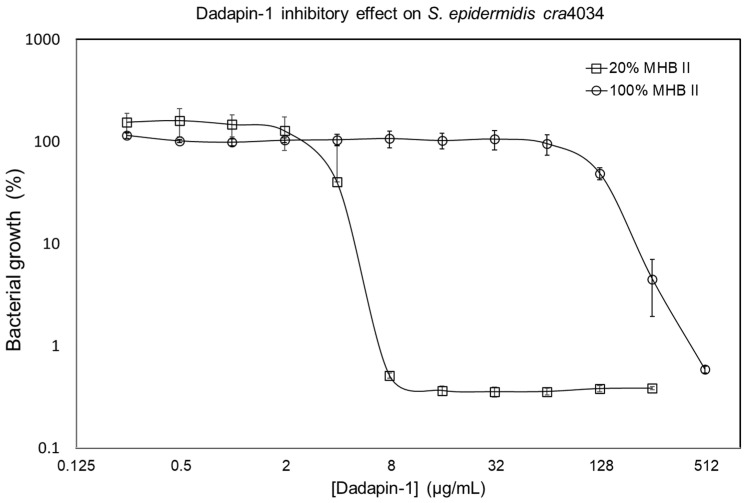
Inhibitory effects of Dadapin-1 on the growth of the *S. epidermidis cra*4034 strain when the inoculum was prepared in undiluted MHB II medium (100% MHB II) and when the inoculum was prepared in 1:5 diluted MHB II medium (20% MHB II). The values of luminescence in RLU were normalized with the reference control equated to 100%. The points represent the mean ± S.D. of triplicated wells from a single experiment. The MIC was determined as the first concentration of Dadapin-1 causing a reduction of at least 1 log (90% inhibition).

The antimicrobial activity of Dadapin-1 showed striking variations depending on the dilution of the medium used to prepare the bacterial inoculum. MHB II is a broth optimized for the growth of a broad range of bacterial species; nonetheless, it was described to contain chemical components capable of neutralizing part of the activity of antimicrobial peptides [17]. This interaction of the undiluted MHB II medium with Dadapin-1 clearly emerged from our data (see Table 1) as, except for coagulase-negative staphylococci (CNS), all the other bacterial strains exhibited MICs greater than the maximal concentration of Dadapin-1 tested. On the contrary, we found that, in diluted culture medium, bacterial strains indicated a pronounced antimicrobial activity of Dadapin-1, with MICs in the range of 3.1–24.9 µM. Nevertheless, under these testing conditions with more limited nutrients, not all strains were capable of significant growth and in a few cases the lack of growth impeded the possibility to measure the MIC values. In fact, for a number of strains (these particularly including CNS species), the poor growth resulted in a low OD (sometimes as low as 0.020 optical density units after subtraction of the blank), thus hindering a correct MIC determination (ND in Table 1). Luminescence made it easier to obtain a MIC determination due to the higher sensitivity of this technique enabling quantification even of a few viable bacterial cells (Figure 1), a broader range of reading linearity as well as because the curve describing the effects of the test substance at different concentrations was usually very steep.

Nonetheless, for *S. lugdunensis cra*4011 and *S. haemolyticus cra*3885, a MIC could not be clearly and rigorously identified even from the curve achieved by luminescence. In order to apply correctly the threshold of a log reduction to discriminate the MIC by means of luminescence, it is necessary that the growth of bacteria over the 24 h is of at least 2 logs, and this was not always the case with diluted MHB II. In many cases, the MIC values estimated by luminescence were closely matching those by OD, particularly for treatments in undiluted broth and, thus, with optimal bacterial growth. However, MIC values tended to be a dilution lower than those determined by luminescence when tested in diluted medium.

Conversely, MIC values obtained by luminescence seemed more robust and consistent. In undiluted MHB II, both *S. aureus* strains and the two gram-negative species showed a MIC greater than 198.9 µM. Both *S. epidermidis* strains exhibited a MIC of 99.4 µM, and the other CNS strains a MIC of 24.9 µM. In diluted MHB II, when determinable, four staphylococcal strains exhibited a MIC of 3.1 µM and one of 6.2 µM. Vice versa, a higher MIC of 12.4 µM was reported for the gram-negative strains except for *P. aeruginosa cra*4010 (MIC = 12.4–24.9 µM).

As far as the MBC was concerned, in undiluted MHB II only for the three CNS species other than *S. epidermidis* was it possible to determine a value within the range of Dadapin-1 concentrations tested (see Table 2).

In diluted MHB II, for staphylococcal strains, the MBC was found to be in the range of 3.1–12.4 µM. Moreover, MBC testing with diluted MHB II was possible even for CNS strains such as *S. lugdunensis cra*4011 and *S. haemolyticus cra*3885 that, under the same experimental conditions, did not allow the determination of a MIC. The MBC for the two strains was, respectively, of 3.1–6.2 µM and 12.4 µM. Among gram-negative strains, the MBC value was in the range of 12.4–49.7 µM.

As far as the inhibition of biofilm formation is concerned (Table 3), the MBIC values observed were generally the same as the MIC values (MBIC = 1× MIC). This was not the case for *S. epidermidis cra*4034 (MBIC = 2× MIC) and for the *P. aeruginosa cra*4010 strain, whose MIC in undiluted broth was greater than 198.9 µM, while the MBIC corresponded to 198.9 µM.

### 2.2. Dadapin-1 Cytotoxicity

**Figure 2:**
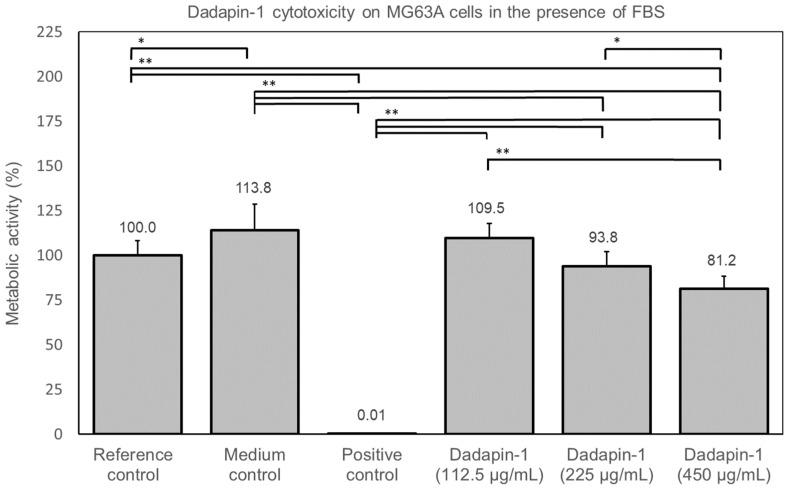
The concentration versus cytotoxicity curve showed that the IC50 was greater than the highest Dadapin-1 concentration tested. Therefore, this bar graph reports just the metabolic activity observed in the MG63 after the treatment with the three highest investigated concentrations of Dadapin-1. The RLU values of the luminescence assay were normalized with respect to the reference control, which was equated to 100%. For the different controls, the bars report the mean ± S.D. of 12 measurements and for Dadapin-1 treatments of 9 measurements from three independent experiments. The data were statistically analyzed by ANOVA followed by Bonferroni/Dunn test. Comparisons are not significant unless *p* < 0.0033. Legend: *, *p* = 0.0004; **, *p* < 0.0001.

The luminescence ATP assay was adopted to investigate the effects of Dadapin-1 on the viability of osteoblastoma MG63 cells. When Dadapin-1 was tested in the presence of FBS, it exhibited an excellent in vitro cytocompatibility. the luminescence assay indicated a just slight decrease in cell metabolism (ATP production) at the two highest concentrations of the peptide. All experimental data, row and elaborated, are reported in extenso in Tables S2–S5. In Figure 2 it is possible to appreciate the low level of toxicity of the Dadapin-1 on MG63 cells. The 6.2% decrease in cell metabolism observed at 89.5 µM (225 µg/mL) of Dadapin-1 was found not to differ significantly from the reference control. Only at 179.0 (450 µg/mL), the antimicrobial peptide determined a slight, but statistically significant, decrease of 18.8% in cell metabolic activity. As expected, the medium control showed a small increase in cell activity with respect to the reference control, while the positive control consisting of 4% Tween-20 produced a substantial reduction in cell viability.

When the test was performed in the absence of serum, Dadapin-1 resulted cytotoxic but only at the highest concentration assayed. Thus, in this case it was possible to attempt to calculate an approximate IC50 value. Ideally, though, for a perfect sigmoidal curve, further concentrations of Dadapin-1, higher than the IC50, should have been tested. Figure S1 reports the curve achieved by Quest Graph™ IC50 Calculator. The IC50 value provided by the online tool provided an IC50 value of 315.4 µg/mL. The Selectivity Index (SI) was calculated as the ratio between IC50 and MIC and was found in the range of 20.2–40.4, 40.4 for CNS species, 10.1 for *E. coli*, and in the range of 5.0–10.1 for *P. aeruginosa*.

**Figure 3:**
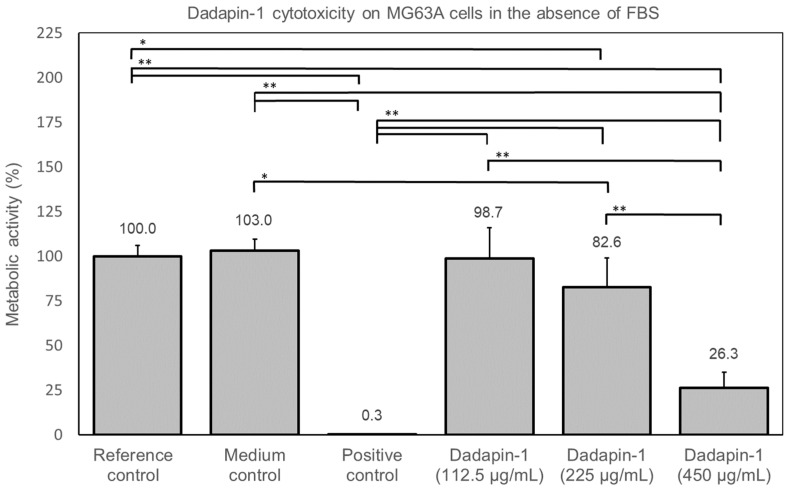
This bar graph reports the metabolic activity observed in the MG63 after the treatment with the three highest investigated concentrations of Dadapin-1 in the absence of FBS. The RLU values of the luminescence assay were normalized with respect to the reference control, which was equated to 100%. For the different controls, the bars report the mean ± S.D. of 8 measurements and for Dadapin-1 of 6 measurements from two independent experiments. The data were statistically analyzed by ANOVA followed by Bonferroni/Dunn test. Comparisons are not significant unless *p* < 0.0033. Legend: *, *p* < 0.0033; **, *p* < 0.0001.

Figure 3 reports the cytotoxicity of the Dadapin-1 when tested in the absence of FBS. It may be noticed that up to the concentration of 225 µg/mL, the peptide causes just a slight reduction in cell activity. All experimental, row and elaborated, data are reported in extenso in Tables S6 and S7.

## 3. Discussion

When testing the properties of candidate antibacterial substances, both EUCAST and CLSI recommend performing the broth dilution method with cation-balanced MHB II (10–12.5 mg/L Mg^2+^ and 20–25 mg/L Ca^2+^) independently on their chemistry and mechanism of action. Nevertheless, there is evidence that MHB may not be a suitable medium for testing cationic peptides as it contains a high quantity of anionic amino acids derived from hydrolyzed casein and other anionic compounds that can interfere with cationic AMP activity [17]. Different studies have shown that, for a given bacterial strain, the MIC values determined using undiluted either MHB or cation-balanced MHB can be several folds higher than the MIC values obtained using diluted solutions of the same broths, anions-depleted MHB (passed through an anion exchange column), or media of completely different formulation (e.g., LM broth, RPMI-1640 medium supplemented with 10% Luria-Bertani broth) [17]. Unfortunately, to present, no suitable medium has been identified that (i) is capable to adequately support the growth of both gram-positive and gram-negative bacteria, (ii) does not quench/mask the antibacterial activity expressed by cationic peptides and, more importantly, closely simulates the behavior of the physiological body fluids, being representative of real in vivo conditions.

This in vitro study on the antimicrobial activity of Dadapin-1 aimed at assessing the MIC on different clinical isolates. The activity of Dadapin-1 was challenged using both undiluted and 20% diluted MHB II. In the early work by Rončević et al. (2019) [15], the antimicrobial activity of Dadapin-1 was originally tested uniquely in diluted MHB II medium and demonstrated very promising properties on the reference strains of five different bacterial species. While in the previous pioneering study, MIC values were assessed by visually inspecting bacterial growth in the microtiter plates, here, two different quantitative techniques were used, and their results compared. Moreover, being interested in investigating the Dadapin-1 antimicrobial activity on the etiological agents causing implant-related infections, the experimental work was conducted on a panel of bacterial clinical isolates from orthopedic infections. Interestingly, all CNS strains showed a measurable MIC even when tested in undiluted MHB II, suggesting a more powerful activity of Dadapin-1 on these species. With 20% MHB II, MICs for the two *S. aureus* clinical isolates were 3.1 µM when assessed by optical reading and 3.1 and 6.2 µM when assessed by luminescence; thus, only marginally higher than the MIC reported in [15] for the *S. aureus* ATCC 29213 reference strain. A MIC of 3.1 µM was determined by luminescence for two strains of *S. epidermidis* and a strain of *S. warneri*, two species that were never tested before with Dadapin-1. For other two relevant CNS species, namely *S. lugdunensis* cra4011 and *S. haemolyticus* cra3885, the interpretation of the inhibition curve was difficult to interpret, mostly due to a relatively poor growth of the bacteria in the diluted broth even in untreated cultures. Concerning gram-negative bacteria such as *E. coli* and *P. aeruginosa*, MIC values, respectively, of 6.2 µM and 6.2–12.4 µM were identified by optical density. They closely approach the values of 8–16 µM for *E. coli* ATCC 25922 and of 8 µM for *P. aeruginosa* ATCC 27853 reported in [15].

As far as the assessment of the MBC is concerned, Dadapin-1 resulted bactericidal on both *S. aureus* strains used in the current study, namely *S. aureus* ATCC25923 and *S. aureus cra*4030, at much greater concentrations than that reported for *S. aureus* ATCC 29213 by Rončević and colleagues, respectively, 12.4 µM and 6.2 µM vs 1 µM. On the contrary, the MBC for *E. coli cra*4038 was 12.4 µM (vs 16–32 µM for *E. coli* ATCC 25922 in [15]), and that of the two *P. aeruginosa* strains of this study was in the range 12.4–49.7 µM (vs 16 µM for *P. aeruginosa* ATCC 27853 in [15]).

These results appear particularly interesting, especially in view of the low level of cytotoxicity observed on M63 cells. Dadapin-1 biological properties were previously investigated by assessing the hemolytic activity in terms of HC_50_. The HC_50_ of the peptide was found to be exceptionally high (670 µM), suggesting a very high SI ranging from 42 to 670 for the five bacterial species tested [15]. Our in vitro cytotoxicity studies on human osteoblast-like MG63 cells prove that in the presence of serum, even at the highest concentration tested (179 µM), Dadapin-1 causes just a slight reduction in metabolic activity (less than 20%), far from reaching an IC_50_. Conversely, in the complete absence of serum, more pronounced effects are observed at the highest concentration tested. This finding could suggest that the presence in serum of proteases could determine at least in part the hydrolysis of the peptide. Alternatively, other components could be capable to sequester or inhibit the activity of AMPs such as Dadapin-1 (e.g., serum albumin) [18,19]. Examining the values of cytotoxicity in Figure 2 and Figure 3, it appears as if the presence of serum could decrease the effects of the peptide, observing at 450 µg/mL roughly the same toxicity seen at 225 µg/mL in the absence of serum. Our observations were conducted on human osteosarcoma MG63 cells. Further investigations would have to be conducted to ascertain if the bland cytotoxic activity observed on these cancer cells is replicated also on normal primary human cells. In peptides with no apparent hemolytic activity, earlier studies have documented different cytotoxic activities depending on the specific cell line used for the tests [20].

D’Este et al. (2017) [21] conducted an in-depth study on the antibacterial properties of a selection of AMPs on clinical isolates from orthopedic infections. The panel of peptides investigated by the authors included the following substances: BMAP-27, BMAP-28, B27(1–18), B28(1–18) and P19. The peptide BMAP-27 and its truncated derivative B27(1-18) emerged as the most promising ones for the prevention of orthopedic infections. Very interestingly, they expressed antibacterial activity against staphylococci with MICs in the range of 0.5–16 µM. When tested on MG63 cells over a period of 24 h, just a restricted concentration range of the peptides was investigated (1.25–10 µM), though BMAP-27 expressed a significant decrease in cell viability at concentrations as low as 10 µM. More interestingly, B27(1–18), its truncated form, showed a much-improved performance. Unfortunately, given the different experimental conditions and the techniques adopted, a direct comparison of the obtained results remains rather difficult.

Overall, Dadapin-1 seems to express considerable potential as antimicrobial substance exhibiting a potent and broad-spectrum activity on some of the most frequent etiologic agents causing implant-related orthopedic infections. Furthermore, concentrations much higher than the MIC can be reached without causing relevant toxicity or significantly affecting the viability of osteoblast-like human cells, when the tests are performed in physiological condition in the presence of serum.

Thus, further studies are ongoing to investigate the possibility of functionalizing the surface of biomaterials used in orthopedics with Dadapin-1, conferring anti-infective properties while preserving optimal biocompatibility. Short lifetime and rapid removal from the circulation represent an advantage, from the point of view of the biocompatibility of AMPs as they do not accumulate in the host tissues, but also, at the same time, a potential point of weakness [14]. Polymeric antimicrobial peptide delivery systems represent a strategy to overcome this limitation [22,23,24]. For this reason, the incorporation of Dadapin-1 in a finely tuned polymeric delivery system to be applied as thin coating on the surface of orthopedic materials will be explored.

## 4. Materials and Methods

### 4.1. Dadapin-1 Antimicrobial Peptide Synthesis

The synthetic peptide Dadapin-1, consisting of 23 amino acids, with aminoacidic sequence GLLRASSKWGRKYYVDLAGCAKA and degree of purity >98%, was purchased from GenicBio Limited (Shanghai, China) as in Rončević et al. (2019) [15] and from Proteogenix Sas (Schiltigheim, France). The peptide was diluted to a concentration of 1 mg/mL in sterile distilled water and aliquots were stored at −80 °C.

### 4.2. Bacterial Strains

For the study of the antibacterial properties of the test peptide, 10 different clinical strains were selected as being representative of the most prevalent etiologic agents causing implant related infections. The strains included a reference strain, namely *Staphylococcus aureus* ATCC^®^25923, and 9 clinical isolates from orthopedic infections: *S. aureus cra*4030, *Staphylococcus epidermidis cra*4034, *S. epidermidis cra*4029, *Staphylococcus warneri cra*3882, *Staphylococcus lugdunensis cra*4011, *Staphylococcus haemolyticus cra*3885, *Escherichia coli cra*4038, *Pseudomonas aeruginosa cra*4010, and *P. aeruginosa cra*4004. All the strains are part of the large collection of clinical isolates of the Laboratory on Pathology of Implant Infections at the IRCCS Rizzoli Orthopedic Institute. Bacterial strains are stored at −80 °C in the internal biorepository “Ceppoteca”, which also includes backup samples of each cryopreserved strain.

### 4.3. Antimicrobial Testing

#### 4.3.1. Minimal Inhibitory Concentration (MIC)

The antibacterial properties of Dadapin-1 were assayed under two distinct conditions of bacterial culture: (i) with bacteria cultured in undiluted Mueller Hinton II Broth (MHB II) and (ii) with bacteria cultured in diluted MHB II. For the MIC test, a few colonies of each bacterial strain, taken from overnight cultures on Mueller Hinton Agar plates (MHA, B19372, Meus, Piove di Sacco, Italy), were resuspended in Mueller Hinton II Broth (MHB II, 24107, Liofilchem, Roseto degli Abruzzi, Italy) either undiluted or at a 20% dilution in water. The concentration of bacteria was adjusted to approximately 10^8^ CFU/mL, as estimated by optical density reading at 625 nm, using a Hewlett Packard G1103A spectrophotometer (Waldbronn, Germany). The bacterial suspension was further diluted to a concentration of 1:100 in 100% or 20% MHB II. Serial 1:2 dilutions of Dadapin-1 were prepared in sterile deionized water starting from a stock solution of approximately 1 mg/mL. Each microtiter plate (96-Well CytoOne^®^ Plate, CC7672-7596, Starlab S.r.l., Milano, Italy) was prepared by adding a volume of 100 µL of inoculum to 100 µL of treatment. Each dilution was assayed in triplicate wells. The final concentration of bacteria was of approximately 5 × 10^5^ CFU/mL, and the range of concentrations of Dadapin-1 was between 500 µg/mL (198.9 µM) and 0.24 µg/mL (0.097 µM) when the tests were performed in 100% MHB II and between 250 µg/mL (99.4 µM) and 0.12 µg/mL (0.049 µM) when the tests were performed in 20% MHB II. The microplates included triplicate wells with 100 µL of sterile distilled water and 100 µL of sterile MHB II for the blank, and 3–6 wells with 100 µL of sterile distilled water and 100 µL of MHB II inoculated with the bacterium for reference control of 100% growth. Two intraplate positive controls were performed in triplicate wells with 100 µL of a 10 mg/mL gentamicin solution (G1272, Sigma Aldrich, Milan, Italy) and with 100 µL of a solution containing 10,000 units/mL of penicillin and 10,000 µg/mL of streptomycin (Penicillin–Streptomycin, 15140-122, Life Technologies, Monza, Italy).

After one day of incubation at 37 °C, the optical density of the plates was read at a wavelength of 600 nm using a Modulus II multifunction plate reader (Turner BioSystems, Sunnyvale, CA, USA). MIC was estimated from the curves obtained by plotting the results of 1 to 4 independent experiments conducted at different times (see Table S1). In addition to the assessment by the optical reading, the MIC was also assessed by luminescence using the kit Bac-Titer Glo Microbial Cell Viability Assay (G8231, Promega Italia S.r.l, Milan, Italy). Following the indication of the producer, 100 µL of each treated culture were transferred to 96-well non-treated plates (236105/237105, Life Technologies, Monza, Italy) and 100 µL of reconstituted solution was added. Relative luminescence units (RLUs) were read by a Modulus II multifunction plate reader (Turner BioSystems, Sunnyvale, CA, USA). In this case, MIC was identified from the curves of growth inhibition as the first concentration leading to a 1-log reduction in bacterial growth (i.e., 90% growth inhibition). A close matching was generally observed between MIC estimated by optical reading and luminescence. Nonetheless, when using 20% MHB II, the growth of some strains was low and optical density reading was not as sensitive as luminescence in detecting the bacterial density.

#### 4.3.2. Minimal Bactericidal Concentration (MBC)

For assessing the MBC, 100 μL of bacterial suspensions were taken from wells corresponding to MIC, 2 × MIC, and 4 × MIC and then plated on MHA plates. After incubation at 37 °C for 1 day, the agar plates were examined for bacterial growth. The MBC was calculated as the concentration causing the growth ≤10 CFU per plate.

#### 4.3.3. Minimal Biofilm Inhibitory Concentration (MBIC)

The quantity of biofilm formation after a day of treatment was assessed by luminescence assay. Once completely removed, any residual bacterial suspension from the microtiter plates was processed as described above; the wells were washed twice with 1× DPBS (14040-091, Life Technologies, Monza, Italy) to remove non-adhered bacteria. Then, a volume of 100 µL DPBS and a volume of 100 µL of reconstituted solution of the Bac-Titer Glo Microbial Cell Viability Assay (G8231, Promega Italia S.r.l, Milan, Italy) was added, following the indications of use of the producer. After 5 min of incubation, a volume of 100 µL was transferred from each well to 96-well non-treated plates for luminescence reading (236105/237105, Life Technologies, Monza, Italy). The luminescence was finally read by a Modulus II multifunction plate reader (Turner BioSystems, Sunnyvale, CA, USA).

### 4.4. Cytotoxicity Testing on MG63 Cells

The cytotoxicity of the Dadapin-1 was assayed on the human osteosarcoma cell line MG63 (ATCC, Rockville, MD, USA). MG63 cells were thawed from a frozen stock and routinely cultured in MEM growth medium (Invitrogen Ltd., Paisley, UK), supplemented with 10% heat-inactivated fetal bovine serum (FBS, Invitrogen Ltd., Paisley, UK), and penicillin/streptomycin (10,000 U/mL penicillin, 10 mg/mL streptomycin, Sigma-Aldrich, Milan, Italy), under standard culture conditions. Cells were regularly sub-cultured using a Trypsin-EDTA solution (Sigma-Aldrich, Milan, Italy) to detach adhered cells.

The effects of Dadapin-1 on MG63 cells were assayed by a bioluminescent ATP assay. Briefly, 96-well tissue-culture treated plates (96-Well Nunclon Delta White, Life Technologies, Monza, Italy) were prepared by adding to each well 100 μL of a suspension of MG63 cells in MEM complete medium at a concentration of 5 × 10^4^ cells/mL. After 1 day of incubation at 37 °C under standard cell culture conditions, 96-well plates were treated with serial dilutions of Dadapin-1. The initial dilution was prepared adding 1 volume of 10× Dulbecco’s Phosphate-Buffered Saline (DPBS, 14200-059, Life Technologies, Monza, Italy) to 9 of the stock solution of Dadapin-1 in water (1 mg/mL) and further dilution were made in 1× DPBS. The final test concentrations were in the range 450–0.22 µg/mL. Triplicate wells were used per each dilution and the test was replicated in 3 independent experiments. The reference control was obtained by treating the cells with 1× DPBS. Further controls used in each plate of the cytotoxicity tests included the medium control, consisting of MEM medium fully supplemented with FBS and antibiotics, and a positive control solution, consisting of 4% Tween-20 (Sigma-Aldrich, Milan, Italy) in 1× DPBS.

After further 24 h of incubation at 37 °C, the 96-well plates were processed as follows: a volume of 100 μL of medium was removed from each well and replaced by a corresponding volume of solution of the CellTiter-Glo 2.0 Assay (Promega, Milano, Italy). Following 10 min of incubation at room temperature in the dark, the plates were processed for luminescence reading by the Modulus II Multifunction Plate Reader (Turner BioSystems, Sunnyvale, CA, USA). After subtracting the blank from all readings, the luminescence values for the treated wells were normalized with the mean of the reference control equated to 100%.

To explore the cytotoxicity in the absence of FBS, further experiments were conducted preparing the 96-well plates seeded with MG63 cells as described above but removing the FBS supplemented medium and washing the wells once with 1× DPBS, just before applying the treatments prepared in medium without serum.

### 4.5. Statistics

The statistical analysis of cytotoxicity data was performed by ANOVA followed by Bonferroni/Dunn test (significance level: 5%) using StatView (version 5.0.1, Sas Institute Inc.). The IC50 value for the Dadapin-1 cytotoxicity in MG63 cells in the absence of FBS was obtained using the Quest Graph™ IC50 Calculator, AAT Bioquest, Inc. (https://www.aatbio.com/tools/ic50-calculator, accessed on 12 November 2022) online resource. The parameter mode of the calculator was set to “Three parameters”.

## 5. Conclusions

The findings from the microbiological characterization confirm a potent antibacterial activity of Dadapin-1 and demonstrate ideal cytocompatibility properties. At the same time, they highlight the need to identify more suitable culture media for testing the antibacterial activity of cationic AMPs on a broader range of bacterial species (included fastidious strains), ensuring optimal growth, absence of polyanionic components at risk of complexation with polycationic peptides, and better simulation of in vivo clinical conditions. In the in vitro cytotoxicity tests, the presence of serum resulted in a slight increase in toxicity in MG63 cancer cells. This observation certainly warrants further investigations to investigate the role of proteases in degrading Dadapin-1 and the possible differences when using human serum instead of bovine serum. Generally, AMPs are known to be rather vulnerable to protease degradation [25]. However, it has also been reported that proteolytic degradation of peptide-based therapeutics may often be misleading, stimulating efforts to stabilize peptides that are relevant only in vitro, but of lower importance in vivo [26].

## Figures and Tables

**Table 1:** MIC values obtained in undiluted and diluted MHB II.

Bacterial Strain	100% MHB II		20% MHB II	
	O.D.	LUM	O.D.	LUM
*S. aureus ATCC25923*	>500 (>198.9)	>500 (>198.9)	7.8 (3.1) *	7.8 (3.1) *
*S. aureus cra*4030	>500 (>198.9)	>500 (>198.9)	7.8 (3.1)	15.6 (6.2)
*S. epidermidis cra*4034	250 (99.4) *	250 (99.4) *	3.9 (1.6)	7.8 (3.1)
*S. epidermidis cra*4029	250 (99.4) *–500 (198.9)	250 (99.4) *	ND	7.8 (3.1)
*S. warneri cra*3882	31.3 (12.4)	62.5 (24.9)	ND	7.8 (3.1)
*S. lugdunensis cra*4011	62.5 (24.9) *	62.5 (24.9) *	ND	ND
*S. haemolyticus cra*3885	62.5 (24.9) *	62.5 (24.9) *	ND	ND
*E. coli cra*4038	>500 (>198.9)	>500 (>198.9)	15.6 (6.2)	31.3 (12.4)
*P. aeruginosa cra*4010	>500 (>198.9)	>500 (>198.9)	15.6 (6.2)–31.3 (12.4) *	31.3 (12.4) *–62.5 (24.9)
*P. aeruginosa cra*4004	>500 (>198.9)	>500 (>198.9)	31.3 (12.4) *	31.3 (12.4) *

MIC values are provided in µg/mL and in µM concentration between brackets. NP, not performed; ND, not determined. MIC could not be determined (likely due to the poor growth of the bacterial strain in diluted MHB II). *, perfect matching between MIC determined by O.D. and by luminescence.

**Table 2:** MBC values obtained in undiluted and diluted MHB II.

Bacterial Strain	100% MHB II	20% MHB II
*S. aureus ATCC25923*	>500 (>198.9)	31.3 (12.4)
*S. aureus cra*4030	>500 (>198.9)	15.6 (6.2)
*S. epidermidis cra*4034	>500 (>198.9)	7.8 (3.1)
*S. epidermidis cra*4029	>500 (>198.9)	31.3 (12.4)
*S. warneri cra*3882	125 (49.7)	7.8 (3.1)
*S. lugdunensis cra*4011	250 (97.8)	7.8 (3.1)–15.6 (6.2)
*S. haemolyticus cra*3885	250 (97.8)	31.3 (12.4)
*E. coli cra*4038	>500 (>198.9)	31.3 (12.4)
*P. aeruginosa cra*4010	>500 (>198.9)	31.3 (12.4)–125 (49.7)
*P. aeruginosa cra*4004	>500 (>198.9)	31.3 (12.4)

MIC values are provided in µg/mL and in µM concentration between brackets.

**Table 3:** MBIC values obtained in undiluted and diluted MHB II.

Bacterial Strain	100% MHB II	20% MHB II
*S. aureus ATCC25923*	>500 (>198.9)	7.8 (3.1)
*S. aureus cra*4030	>500 (>198.9)	15.6 (6.2)
*S. epidermidis cra*4034	500 (198.9)	7.8 (3.1)
*S. epidermidis cra*4029	250 (99.4)	7.8 (3.1)
*S. warneri cra*3882	62.5 (24.9)	3.9 (1.6)
*S. lugdunensis cra*4011	62.5 (24.9)	ND
*S. haemolyticus cra*3885	62.5 (24.9)	ND
*E. coli cra*4038	>500 (>198.9)	31.3 (12.4)
*P. aeruginosa cra*4010	500 (198.9)	31.3 (12.4)–62.5 (24.9)
*P. aeruginosa cra*4004	>500 (>198.9)	31.3 (12.4)

MIC values are provided in µg/mL and in µM concentration between brackets.

## Data Availability

Information on the number of experiments performed for the MIC determination by optical density and luminescence is reported in detail in Table S1. Moreover, all row and elaborated data from the cytotoxicity study are reported in extenso in the Supplementary Materials (Tables S2–S5).

## Supplementary Materials

The following supporting information can be downloaded at: https://www.mdpi.com/article/10.3390/ijms24010779/s1.
