# Supplementary material for: Antibacterial Activity on Orthopedic Clinical Isolates and Cytotoxicity of the Antimicrobial Peptide Dadapin-1
Source: Int J Mol Sci. 2023 Jan 2;24(1):779. doi: 10.3390/ijms24010779 (PMC9821071; doi:10.3390/ijms24010779)
Supplement: Supplementary file 1 [file ijms-24-00779-s001.zip › ijms-2107301-supplementary.pdf]

Table S1. Number of replicated experiments with triplicated measurements performed for the evaluation of MIC

Species	Strain	Origin	Medium	OD		RLU	
				MIC (μg/mL)	Replicated tests	MIC (μg/mL)	Replicated tests
S. aureus	ATCC25923	Reference strain	MHB II 100%	>500	2 times (n=6)	>500	1 time (n=3)
			MHB 20%	7.8	2 time (n=6)	7.8	1 time (n=3)
	*cra*4030	Clinical isolate (IOR)	MHB II 100%	>500	2 times (n=6)	>500	1 time (n=3)
			MHB 20%	7.8	1 time (n=3)	15.6	1 time (n=3)
S. epidermidis	*cra*4034	Clinical isolate (IOR)	MHB II 100%	250	4 times (n=12)	250	1 time (n=3)
			MHB 20%	3.9	1 time (n=3)	7.8	1 time (n=3)
	*cra*4029	Clinical isolate (IOR)	MHB II 100%	250-500	2 times (n=6)	250	1 time (n=3)
			MHB 20%	ND	2 times (n=6)	7.8	2 times (n=6)
S. warneri	*cra*3882	Clinical isolate (IOR)	MHB II 100%	31.3	1 time (n=3)	62.5	1 time (n=3)
			MHB 20%	ND	1 time (n=3)	7.8	1 time (n=3)
S. lugdunensis	*cra*4011	Clinical isolate (IOR)	MHB II 100%	62.5	1 time (n=3)	62.5	1 time (n=3)
			MHB 20%	ND	2 times (n=6)	ND	2 times (n=6)
S. haemolyticus	*cra*3885	Clinical isolate (IOR)	MHB II 100%	62.5	1 time (n=3)	62.5	1 time (n=3)
			MHB 20%	ND	1 time (n=3)	ND	1 time (n=3)
E. coli	*cra*4038	Clinical isolate (IOR)	MHB II 100%	>500	1 time (n=3)	>500	1 time (n=3)
			MHB 20%	15.6	1 time (n=3)	31.3	1 time (n=3)
P. aeruginosa	*cra*4010	Clinical isolate (IOR)	MHB II 100%	>500	3 times (n=9)	>500	1 time (n=3)
			MHB 20%	15.6-31.3*	3 times (n=9)	31.3-62.5**	3 times (n=9)
	*cra*4004	Clinical isolate (IOR)	MHB II 100%	>500	1 time (n=3)	>500	1 time (n=3)
			MHB 20%	31.3	1 time (n=3)	31.3	1 time (n=3)

* Two experiments provided a MIC of 15.6 and an experiment of 31.3 μg/mL; ** Two experiments provided a MIC of 31.3 and an experiment of 62.5 μg/mL.

Table S2. Experimental data from the 3 independent experiments on the cytotoxicity of Dadapin-1 in the presence of FBS: luminescence readings at different Dadapin-1 concentrations.

Well	Row	[Dadapin-1](ug/mL)	Date	Sample (RLU)	Blank (RLU)	Sample-Blank	Cell activity (%)
1	A	450.0	23.09.2022	7699850	449.5	7699400.5	81.08
1	B	450.0	23.09.2022	8156380	449.5	8155930.5	85.89
1	C	450.0	23.09.2022	8657350	449.5	8656900.5	91.16
2	A	225.0	23.09.2022	7874880	449.5	7874430.5	82.92
2	B	225.0	23.09.2022	8659550	449.5	8659100.5	91.19
2	C	225.0	23.09.2022	9658150	449.5	9657700.5	101.70
3	A	112.5	23.09.2022	9978880	449.5	9978430.5	105.08
3	B	112.5	23.09.2022	10593900	449.5	10593450.5	111.56
3	C	112.5	23.09.2022	11188100	449.5	11187650.5	117.81
4	A	56.3	23.09.2022	10093000	449.5	10092550.5	106.28
4	B	56.3	23.09.2022	10964300	449.5	10963850.5	115.46
4	C	56.3	23.09.2022	11611400	449.5	11610950.5	122.27
5	A	28.1	23.09.2022	9981410	449.5	9980960.5	105.11
5	B	28.1	23.09.2022	10859300	449.5	10858850.5	114.35
5	C	28.1	23.09.2022	10607600	449.5	10607150.5	111.70
6	A	14.1	23.09.2022	10469000	449.5	10468550.5	110.24
6	B	14.1	23.09.2022	10034900	449.5	10034450.5	105.67
6	C	14.1	23.09.2022	10681600	449.5	10681150.5	112.48
7	A	7.0	23.09.2022	10699600	449.5	10699150.5	112.67
7	B	7.0	23.09.2022	10273600	449.5	10273150.5	108.18
7	C	7.0	23.09.2022	10782300	449.5	10781850.5	113.54
8	A	3.5	23.09.2022	9820690	449.5	9820240.5	103.41
8	B	3.5	23.09.2022	10556900	449.5	10556450.5	111.17
8	C	3.5	23.09.2022	11321400	449.5	11320950.5	119.22

9	A	1.8	23.09.2022	8409810	449.5	8409360.5	88.56
9	B	1.8	23.09.2022	9987160	449.5	9986710.5	105.17
9	C	1.8	23.09.2022	11129000	449.5	11128550.5	117.19
10	A	0.9	23.09.2022	10197100	449.5	10196650.5	107.38
10	B	0.9	23.09.2022	10609700	449.5	10609250.5	111.72
10	C	0.9	23.09.2022	10895100	449.5	10894650.5	114.73
11	A	0.4	23.09.2022	9775350	449.5	9774900.5	102.94
11	B	0.4	23.09.2022	10654300	449.5	10653850.5	112.19
11	C	0.4	23.09.2022	10856900	449.5	10856450.5	114.33
12	A	0.2	23.09.2022	9709620	449.5	9709170.5	102.24
12	B	0.2	23.09.2022	9666490	449.5	9666040.5	101.79
12	C	0.2	23.09.2022	9870080	449.5	9869630.5	103.93
1	A	450	28.09.2022	10232900	635.5	10232264.5	81.63
1	B	450	28.09.2022	11395800	635.5	11395164.5	90.90
1	C	450	28.09.2022	9661990	635.5	9661354.5	77.07
2	A	225	28.09.2022	12632400	635.5	12631764.5	100.77
2	B	225	28.09.2022	13264400	635.5	13263764.5	105.81
2	C	225	28.09.2022	12324400	635.5	12323764.5	98.31
3	A	112.5	28.09.2022	14125700	635.5	14125064.5	112.68
3	B	112.5	28.09.2022	15375600	635.5	15374964.5	122.65
3	C	112.5	28.09.2022	14339200	635.5	14338564.5	114.38
4	A	56.25	28.09.2022	15047800	635.5	15047164.5	120.04
4	B	56.25	28.09.2022	15152600	635.5	15151964.5	120.87
4	C	56.25	28.09.2022	14575400	635.5	14574764.5	116.27
5	A	28.1	28.09.2022	14721200	635.5	14720564.5	117.43
5	B	28.1	28.09.2022	15346600	635.5	15345964.5	122.42
5	C	28.1	28.09.2022	14084900	635.5	14084264.5	112.36
6	A	14.1	28.09.2022	14600100	635.5	14599464.5	116.47

6	B	14.1	28.09.2022	15379400	635.5	15378764.5	122.68
6	C	14.1	28.09.2022	14640800	635.5	14640164.5	116.79
7	A	7.0	28.09.2022	14829000	635.5	14828364.5	118.29
7	B	7.0	28.09.2022	14630200	635.5	14629564.5	116.71
7	C	7.0	28.09.2022	14585500	635.5	14584864.5	116.35
8	A	3.5	28.09.2022	14585100	635.5	14584464.5	116.35
8	B	3.5	28.09.2022	14418500	635.5	14417864.5	115.02
8	C	3.5	28.09.2022	14513900	635.5	14513264.5	115.78
9	A	1.8	28.09.2022	14300700	635.5	14300064.5	114.08
9	B	1.8	28.09.2022	14722800	635.5	14722164.5	117.44
9	C	1.8	28.09.2022	14296000	635.5	14295364.5	114.04
10	A	0.9	28.09.2022	14952700	635.5	14952064.5	119.28
10	B	0.9	28.09.2022	15397900	635.5	15397264.5	122.83
10	C	0.9	28.09.2022	15109200	635.5	15108564.5	120.53
11	A	0.4	28.09.2022	14898500	635.5	14897864.5	118.85
11	B	0.4	28.09.2022	15016800	635.5	15016164.5	119.79
11	C	0.4	28.09.2022	14191400	635.5	14190764.5	113.21
12	A	0.2	28.09.2022	14147700	635.5	14147064.5	112.86
12	B	0.2	28.09.2022	13961000	635.5	13960364.5	111.37
12	C	0.2	28.09.2022	14535300	635.5	14534664.5	115.95
1	A	450	7.10.2022	9681560	640.0	9680920.0	75.67
1	B	450	7.10.2022	9685180	640.0	9684540.0	75.70
1	C	450	7.10.2022	9121050	640.0	9120410.0	71.29
2	A	225	7.10.2022	11618200	640.0	11617560.0	90.81
2	B	225	7.10.2022	10761800	640.0	10761160.0	84.11
2	C	225	7.10.2022	11327500	640.0	11326860.0	88.54
3	A	112.5	7.10.2022	13187500	640.0	13186860.0	103.07
3	B	112.5	7.10.2022	12799300	640.0	12798660.0	100.04

3	C	112.5	7.10.2022	12620700	640.0	12620060.0	98.64
4	A	56.25	7.10.2022	13432300	640.0	13431660.0	104.99
4	B	56.25	7.10.2022	13311200	640.0	13310560.0	104.04
4	C	56.25	7.10.2022	13096900	640.0	13096260.0	102.37
5	A	28.1	7.10.2022	13934500	640.0	13933860.0	108.91
5	B	28.1	7.10.2022	13444300	640.0	13443660.0	105.08
5	C	28.1	7.10.2022	13338600	640.0	13337960.0	104.26
6	A	14.1	7.10.2022	13784100	640.0	13783460.0	107.74
6	B	14.1	7.10.2022	13382800	640.0	13382160.0	104.60
6	C	14.1	7.10.2022	13427600	640.0	13426960.0	104.95
7	A	7.0	7.10.2022	14397900	640.0	14397260.0	112.54
7	B	7.0	7.10.2022	13485900	640.0	13485260.0	105.41
7	C	7.0	7.10.2022	13216800	640.0	13216160.0	103.30
8	A	3.5	7.10.2022	13518100	640.0	13517460.0	105.66
8	B	3.5	7.10.2022	12980200	640.0	12979560.0	101.45
8	C	3.5	7.10.2022	13139300	640.0	13138660.0	102.70
9	A	1.8	7.10.2022	13590500	640.0	13589860.0	106.22
9	B	1.8	7.10.2022	14156200	640.0	14155560.0	110.65
9	C	1.8	7.10.2022	14116000	640.0	14115360.0	110.33
10	A	0.9	7.10.2022	13584900	640.0	13584260.0	106.18
10	B	0.9	7.10.2022	14194200	640.0	14193560.0	110.94
10	C	0.9	7.10.2022	13479100	640.0	13478460.0	105.35
11	A	0.4	7.10.2022	13271200	640.0	13270560.0	103.73
11	B	0.4	7.10.2022	13912700	640.0	13912060.0	108.74
11	C	0.4	7.10.2022	14556200	640.0	14555560.0	113.77
12	A	0.2	7.10.2022	12879300	640.0	12878660.0	100.67
12	B	0.2	7.10.2022	14201600	640.0	14200960.0	111.00
12	C	0.2	7.10.2022	13729000	640.0	13728360.0	107.31

Table S3. Experimental data from the 3 independent experiments on the cytotoxicity of Dadapin-1 in the presence of FBS: luminescence readings of the controls

1	G	**Reference Control**	23.09.2022	9232510	449.5	9232060.5	97.22
2	G		23.09.2022	9668150	449.5	9667700.5	101.81
3	G		23.09.2022	9558410	449.5	9557960.5	100.65
4	G		23.09.2022	9526830	449.5	9526380.5	100.32
1	G		28.09.2022	9842530	635.5	9841894.5	78.51
2	G		28.09.2022	13045300	635.5	13044664.5	104.06
3	G		28.09.2022	13870600	635.5	13869964.5	110.65
4	G		28.09.2022	13385600	635.5	13384964.5	106.78
1	G		7.10.2022	12171800	640.0	12171160.0	95.14
2	G		7.10.2022	12917700	640.0	12917060.0	100.97
3	G		7.10.2022	12472000	640.0	12471360.0	97.48
4	G		7.10.2022	13615200	640.0	13614560.0	106.42
5	G	**Medium Control**	23.09.2022	9675150	449.5	9674700.5	101.88
6	G		23.09.2022	10516600	449.5	10516150.5	110.74
7	G		23.09.2022	9934840	449.5	9934390.5	104.62
8	G		23.09.2022	9892810	449.5	9892360.5	104.17
5	G		28.09.2022	12108200	635.5	12107564.5	96.59
6	G		28.09.2022	13420900	635.5	13420264.5	107.06
7	G		28.09.2022	13309800	635.5	13309164.5	106.17
8	G		28.09.2022	12927600	635.5	12926964.5	103.12
5	G		7.10.2022	16924900	640.0	16924260.0	132.29
6	G		7.10.2022	16413600	640.0	16412960.0	128.29
7	G		7.10.2022	17477300	640.0	17476660.0	136.61
8	G		7.10.2022	17221800	640.0	17221160.0	134.61

9	G	**Positive Control**	23.09.2022	2858	449.5	2408.8	0.03
10	G		23.09.2022	696	449.5	246.5	0.00
11	G		23.09.2022	648	449.5	198.5	0.00
12	G		23.09.2022	1038	449.5	588.5	0.01
9	G		28.09.2022	4319	635.5	3683.2	0.03
10	G		28.09.2022	1680	635.5	1044.6	0.01
11	G		28.09.2022	1594	635.5	958.6	0.01
12	G		28.09.2022	1488	635.5	852.6	0.01
9	G		7.10.2022	5411	640.0	4771.2	0.04
10	G		7.10.2022	1486	640.0	846.1	0.01
11	G		7.10.2022	1518	640.0	878.1	0.01
12	G		7.10.2022	1646	640.0	1006.1	0.01

Table S4. Experimental data from the 3 independent experiments on the cytotoxicity of Dadapin-1 in the presence of FBS: summary table with statistics from all three experiments.

Dadapin-1 [µg/mL]	% CELL ACTIVITY		RLU	
	Mean (%)	S.D.	Mean (RLU)	S.D.
450.0	81.2	7.0	9365209.4	1115837.8
225.0	93.8	8.1	10901789.4	1838778.6
112.5	109.5	8.3	12689300.5	1811593.1
56.3	112.5	8.0	13031080.5	1806712.9
28.1	111.3	6.2	12923692.8	1942778.6
14.1	111.3	6.3	12932791.6	2012095.6
7.0	111.9	5.2	12988402.8	1883922.9
3.5	110.1	6.8	12760990.5	1784836.4
1.8	109.3	8.9	12744777.2	2300305.1
0.9	113.2	6.5	13157191.6	2054960.8
0.4	111.9	5.9	13014241.6	2028183.2
0.2	107.5	5.6	12521657.2	2129129.5

Table S5. Experimental data from the 3 independent experiments on the cytotoxicity of Dadapin-1 in the presence of FBS: cell metabolic activity for the treatments plotted in **Figure 2.**

	Mean (% cell activity)	S.D.
Reference control	100.0	8.1
Medium control	113.8	14.6
Positive control	0.01	0.01
Dadapin-1 (112.5 µg/mL)	109.5	8.3
Dadapin-1 (225 µg/mL)	93.8	8.1
Dadapin-1 (450 µg/mL)	81.2	7.0

Table S6. Experimental data from the 2 independent experiments on the cytotoxicity of Dadapin-1 in the absence of FBS: luminescence readings at different Dadapin-1 concentrations.

Well	Row	[Dadapin-1](ug/mL)	Data	Sample (RLU)	Blank (RLU)	Sample-Blank	Cell activity (%)
1	A	450.0	21.12.2022	2235950	311.5	2235638.5	22.18
1	B	450.0	21.12.2022	1748910	311.5	1748598.5	17.35
1	C	450.0	21.12.2022	1712860	311.5	1712548.5	16.99
2	A	225.0	21.12.2022	6948510	311.5	6948198.5	68.93
2	B	225.0	21.12.2022	6857870	311.5	6857558.5	68.03
2	C	225.0	21.12.2022	6678080	311.5	6677768.5	66.25
3	A	112.5	21.12.2022	8462910	311.5	8462598.5	83.95
3	B	112.5	21.12.2022	8595040	311.5	8594728.5	85.26
3	C	112.5	21.12.2022	8122820	311.5	8122508.5	80.58
4	A	56.3	21.12.2022	9532850	311.5	9532538.5	94.57
4	B	56.3	21.12.2022	9300780	311.5	9300468.5	92.26
4	C	56.3	21.12.2022	9165660	311.5	9165348.5	90.92
5	A	28.1	21.12.2022	10000300	311.5	9999988.5	99.20
5	B	28.1	21.12.2022	9965060	311.5	9964748.5	98.85
5	C	28.1	21.12.2022	9112850	311.5	9112538.5	90.40
6	A	14.1	21.12.2022	9570010	311.5	9569698.5	94.94
6	B	14.1	21.12.2022	9114950	311.5	9114638.5	90.42
6	C	14.1	21.12.2022	9003150	311.5	9002838.5	89.31
7	A	7.0	21.12.2022	9081670	311.5	9081358.5	90.09
7	B	7.0	21.12.2022	9497790	311.5	9497478.5	94.22
7	C	7.0	21.12.2022	8848810	311.5	8848498.5	87.78
8	A	3.5	21.12.2022	9028390	311.5	9028078.5	89.56
8	B	3.5	21.12.2022	9891390	311.5	9891078.5	98.12

8	C	3.5	21.12.2022	9003810	311.5	9003498.5	89.32
9	A	1.8	21.12.2022	8922210	311.5	8921898.5	88.51
9	B	1.8	21.12.2022	9354290	311.5	9353978.5	92.80
9	C	1.8	21.12.2022	8880370	311.5	8880058.5	88.09
10	A	0.9	21.12.2022	9008300	311.5	9007988.5	89.36
10	B	0.9	21.12.2022	9138000	311.5	9137688.5	90.65
10	C	0.9	21.12.2022	8283040	311.5	8282728.5	82.17
11	A	0.4	21.12.2022	8319240	311.5	8318928.5	82.53
11	B	0.4	21.12.2022	9218890	311.5	9218578.5	91.45
11	C	0.4	21.12.2022	8251220	311.5	8250908.5	81.85
12	A	0.2	21.12.2022	8091570	311.5	8091258.5	80.27
12	B	0.2	21.12.2022	9055360	311.5	9055048.5	89.83
12	C	0.2	21.12.2022	7982240	311.5	7981928.5	79.18
1	A	450	23.12.2022	2850230	237.0	2849993.0	37.21
1	B	450	23.12.2022	2400650	237.0	2400413.0	31.34
1	C	450	23.12.2022	2521500	237.0	2521263.0	32.92
2	A	225	23.12.2022	7600970	237.0	7600733.0	99.25
2	B	225	23.12.2022	7320680	237.0	7320443.0	95.59
2	C	225	23.12.2022	7492800	237.0	7492563.0	97.83
3	A	112.5	23.12.2022	8604410	237.0	8604173.0	112.35
3	B	112.5	23.12.2022	8607640	237.0	8607403.0	112.39
3	C	112.5	23.12.2022	9030690	237.0	9030453.0	117.91
4	A	56.25	23.12.2022	9443670	237.0	9443433.0	123.31
4	B	56.25	23.12.2022	10232300	237.0	10232063.0	133.60
4	C	56.25	23.12.2022	9876340	237.0	9876103.0	128.96
5	A	28.1	23.12.2022	9746920	237.0	9746683.0	127.27
5	B	28.1	23.12.2022	10303300	237.0	10303063.0	134.53
5	C	28.1	23.12.2022	10467300	237.0	10467063.0	136.67

6	A	14.1	23.12.2022	9515940	237.0	9515703.0	124.25
6	B	14.1	23.12.2022	10364200	237.0	10363963.0	135.33
6	C	14.1	23.12.2022	9964630	237.0	9964393.0	130.11
7	A	7.0	23.12.2022	9825840	237.0	9825603.0	128.30
7	B	7.0	23.12.2022	10225600	237.0	10225363.0	133.52
7	C	7.0	23.12.2022	10528500	237.0	10528263.0	137.47
8	A	3.5	23.12.2022	9719500	237.0	9719263.0	126.91
8	B	3.5	23.12.2022	10339900	237.0	10339663.0	135.01
8	C	3.5	23.12.2022	9724780	237.0	9724543.0	126.98
9	A	1.8	23.12.2022	9359080	237.0	9358843.0	122.20
9	B	1.8	23.12.2022	9537550	237.0	9537313.0	124.53
9	C	1.8	23.12.2022	10311300	237.0	10311063.0	134.63
10	A	0.9	23.12.2022	9709820	237.0	9709583.0	126.78
10	B	0.9	23.12.2022	9698620	237.0	9698383.0	126.63
10	C	0.9	23.12.2022	9945860	237.0	9945623.0	129.86
11	A	0.4	23.12.2022	9300720	237.0	9300483.0	121.44
11	B	0.4	23.12.2022	9263040	237.0	9262803.0	120.95
11	C	0.4	23.12.2022	9701410	237.0	9701173.0	126.67
12	A	0.2	23.12.2022	9286840	237.0	9286603.0	121.26
12	B	0.2	23.12.2022	10622500	237.0	10622263.0	138.70
12	C	0.2	23.12.2022	9802870	237.0	9802633.0	128.00

Table S7. Experimental data from the 2 independent experiments on the cytotoxicity of Dadapin-1 in the absence of FBS: luminescence readings of the controls.

**Reference Control**	21.12.2022	10219300	311.5	10218988.5	101.38	
	21.12.2022	10613200	311.5	10612888.5	105.28	
	21.12.2022	9296840	311.5	9296528.5	92.23	
	21.12.2022	10192900	311.5	10192588.5	101.11	100.00
	23.12.2022	6833410	237.0	6833173.0	89.22	
	23.12.2022	7700940	237.0	7700703.0	100.55	
	23.12.2022	7956100	237.0	7955863.0	103.88	
	23.12.2022	8144710	237.0	8144473.0	106.34	100.00
**Medium Control**	21.12.2022	9813790	311.5	9813478.5	97.35	
	21.12.2022	9776870	311.5	9776558.5	96.99	
	21.12.2022	10479000	311.5	10478688.5	103.95	
	21.12.2022	9328500	311.5	9328188.5	92.54	97.71
	23.12.2022	8272570	237.0	8272333.0	108.01	
	23.12.2022	8018250	237.0	8018013.0	104.69	
	23.12.2022	8502740	237.0	8502503.0	111.02	
	23.12.2022	8370250	237.0	8370013.0	109.29	108.25
**Positive Control**	21.12.2022	31587	311.5	31275.4	0.31	
	21.12.2022	30406	311.5	30094.4	0.30	
	21.12.2022	23300	311.5	22988.7	0.23	
	21.12.2022	16287	311.5	15975.4	0.16	0.25
	23.12.2022	39213	237.0	38975.9	0.51	
	23.12.2022	19640	237.0	19402.8	0.25	
	23.12.2022	16553	237.0	16316.2	0.21	
	23.12.2022	13161	237.0	12924.1	0.17	0.29

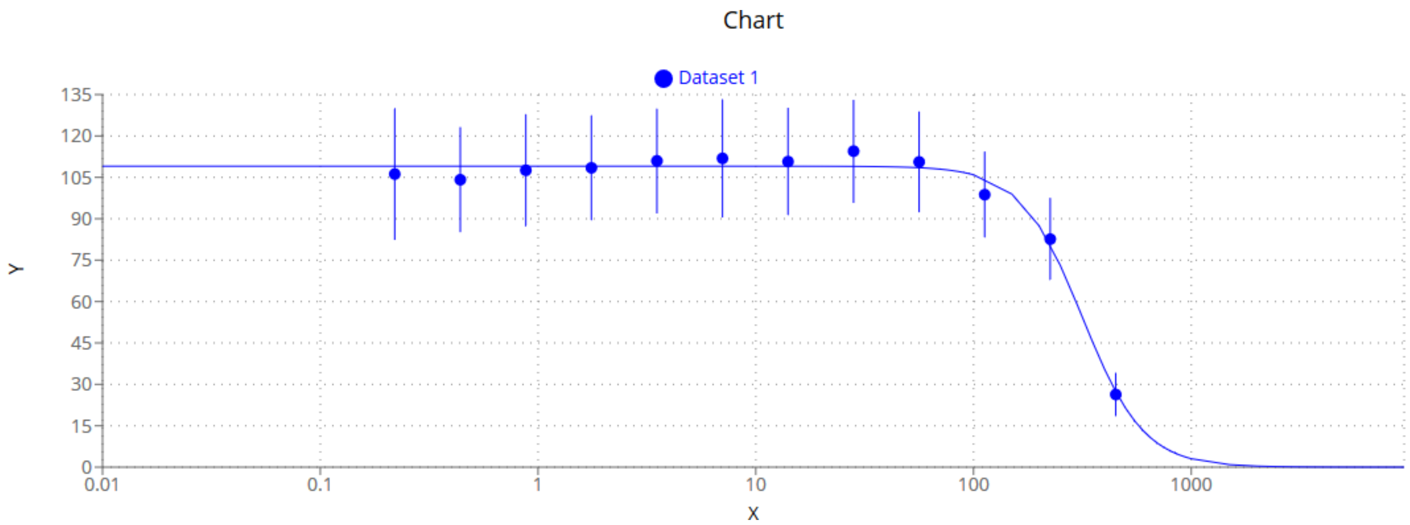

Figure S1. This chart shows the regression curve achieved by Quest Graph™ IC50 Calculator with the cytotoxicity data obtained testing Dadapin-1 on MG63 cells in the absence of FBS. On the X-axis the Dadapin-1 concentration values are reported in µg/mL, while the values on the Y-axis represent the percentage of metabolic activity with respect to the Reference control equated to 100%.
